# Supplementary material for: Adult-born neurons immature during learning are necessary for remote memory reconsolidation in rats
Source: Nat Commun. 2021 Mar 19;12:1778. doi: 10.1038/s41467-021-22069-4 (PMC7979763; doi:10.1038/s41467-021-22069-4)
Supplement: Supplementary file 1 — Supplementary information [file 41467_2021_22069_MOESM1_ESM.pdf]

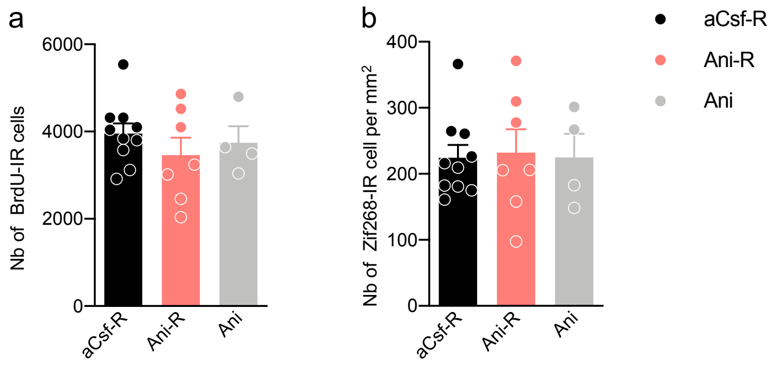

Fig S1. a) Number of BrdU-IR cells in the Dentate Gyrus (DG): the number of BrdU-IR cells was similar between the trained groups(aCsf-R, n=10, Ani-R, n=7, Ani, n=4). **b)** Zif268 expression in the whole DG. The DG activation was similar among the trained groups. All data shown are mean ± s.e.m. For statistical details, see table S1.

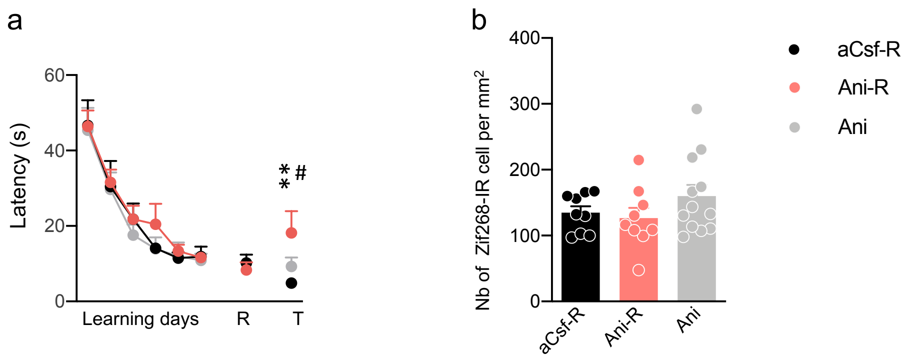

Fig S2. Blocking protein synthesis after spatial memory reactivation impairs remote memory reconsolidation. a) Latency to find the platform during training and to first cross the position of the platform during the reactivation and test trials. Memory performances of Ani-R (n=12) rats were impaired compared to those of aCsf-R (n=12) rats during the test (T) (Tukey's test # p=0.043) and compared to their performances at the reactivation trial (R) (Tukey's test **p<0.01). **b)** Zif268 expression in the whole Dentate Gyrus (DG). The DG activation was similar among the trained groups(aCsf-R n=9, Ani-R n=9, Ani n=12). All data shown are mean ± s.e.m. For statistical details, see table S1.

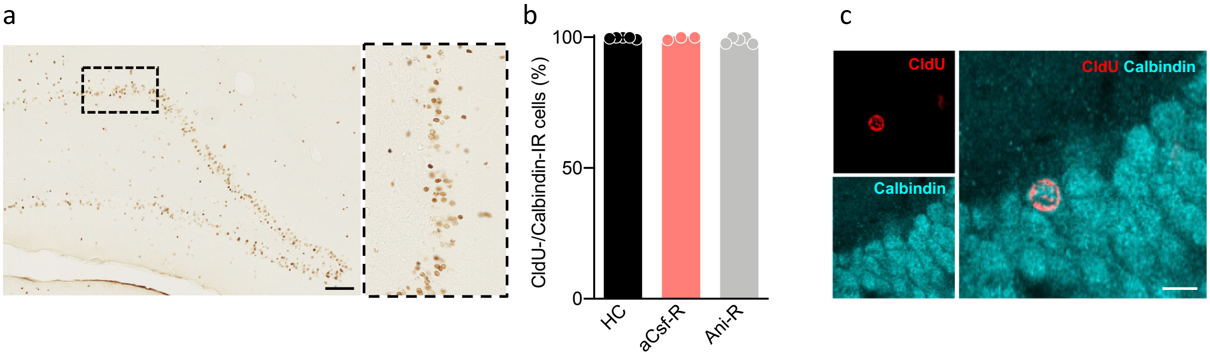

Fig S3: Phenotype of developmentally-generated cells. a) Illustration of CldU-IR cells in the dentate gyrus : Example shown is a representative of a total of >240 dentate gyrus from 30 rats (scale bar 100µm) **b)** Percentage of CldU-IR cells expressing Calbindin (HC: 99.2 ± 0.15, n=5; aCsf-R: 99.7 ± 0.33, n=3; Ani-R: 98.8 ± 0.55, n=5). There was no difference between groups. **c)** Confocal illustration showing CldU-IR cells (red) coexpressing the neuronal marker Calbindin (blue). All data shown are mean ± s.e.m. Bar scale 10 µm.

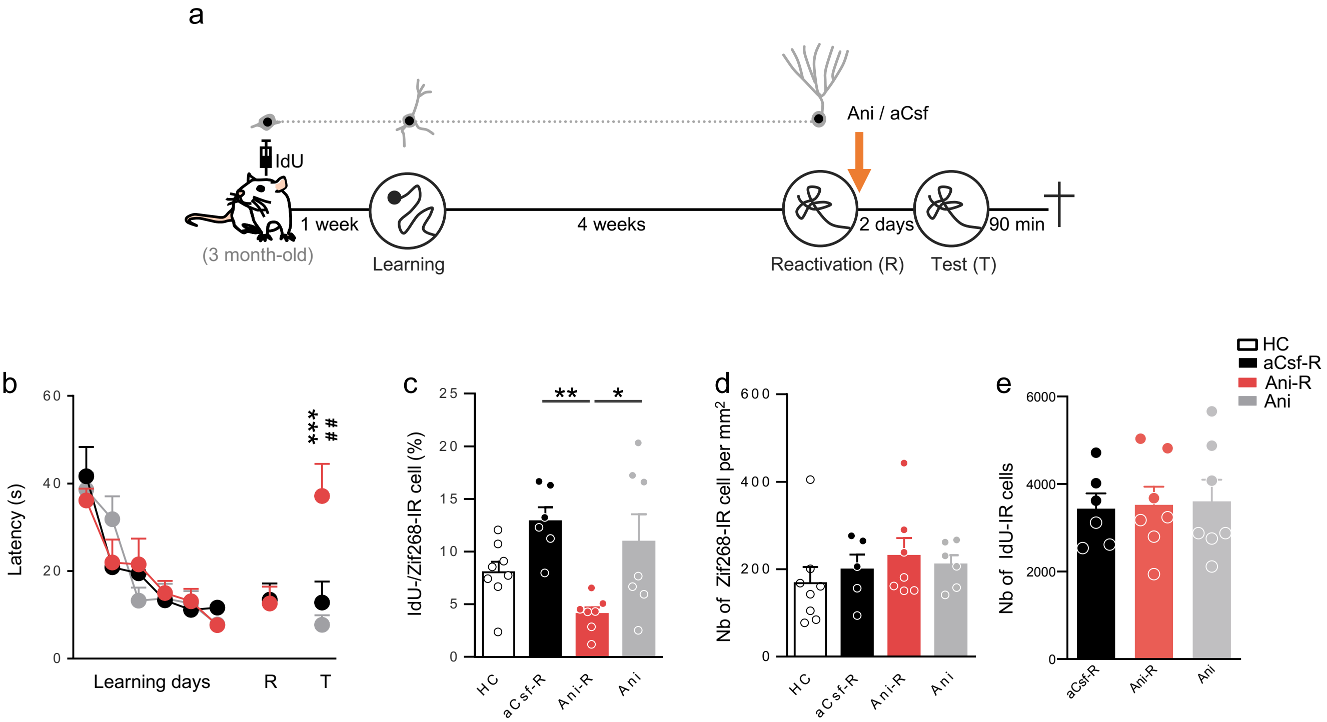

Fig S4. The effect of blocking memory reconsolidation on adult-born neurons retrieval-induced activation does not depend on the age of the animal. a) Experimental protocol: 3 month-old rats were injected with IdU one week before MWM training. Rats were trained for 6 days and memory was reactivated 4 weeks later. Immediately after reactivation rats were injected (icv) with anisomycin (Ani-R, n=7) or with aCsf (aCsf-R, n=6). A group of rats received anisomycin but without the reactivation session (Ani, n=7). Memory was tested 2 days later and rats were sacrificed 90 min after the test. **b)** Latency to find the platform during training and to first cross the position of the platform during the reactivation and test trials. Memory performances of Ani-R rats were impaired compared to those of aCsf-R rat during the test (Tukey's test ##p<0.01) and compared to their performances at the reactivation trial (Tukey's test ***p<0.001). **c)** Zif268 expression in IdU-IR cells. Percentage of expression was higher in the aCsf-R group compared to that of control home cage (HC) rats (n=8) and to that of Ani-R rats (Tukey's test *p<0.05, **p<0,01) but not different than that of Ani rats. **d)** Zif268 expression in the whole Dentate Gyrus (DG). The DG activation was similar between groups. **e)** Number of IdU-IR cells in the DG. The number of IdU-IR cells was similar between groups. All data shown are mean ± s.e.m. For statistical details, see table S1.

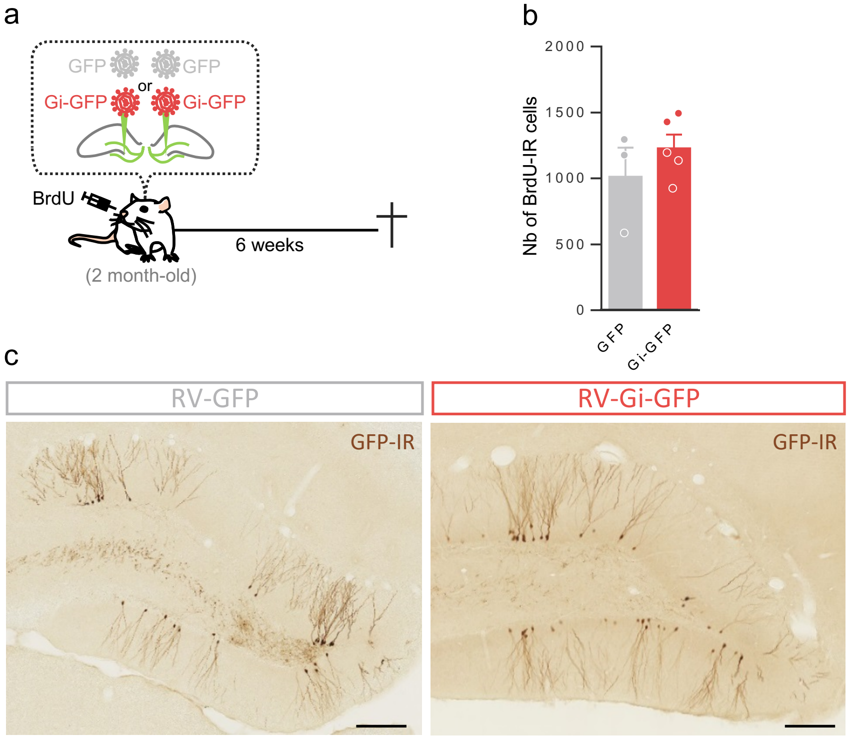

Fig S5. RV infusions do not impact adult neurogenesis. a) Experimental protocol: Two-month old rats were injected with BrdU and injected bilaterally into the DG with the retrovirus GFP-Gi (n=5) or with its control, the GFP-RV (n=3). Six weeks later they were sacrificed. **b)** Adult-born neurons survival. There was no difference between groups (Two-tailed T-test t_6=1.028, p=0.3437). **c)** Illustration of RV-labelled cells in the DG. Example shown is a representative of a total of >64 dentate gyrus from 8 rats (scale=100µm). For statistical details, see table S1.

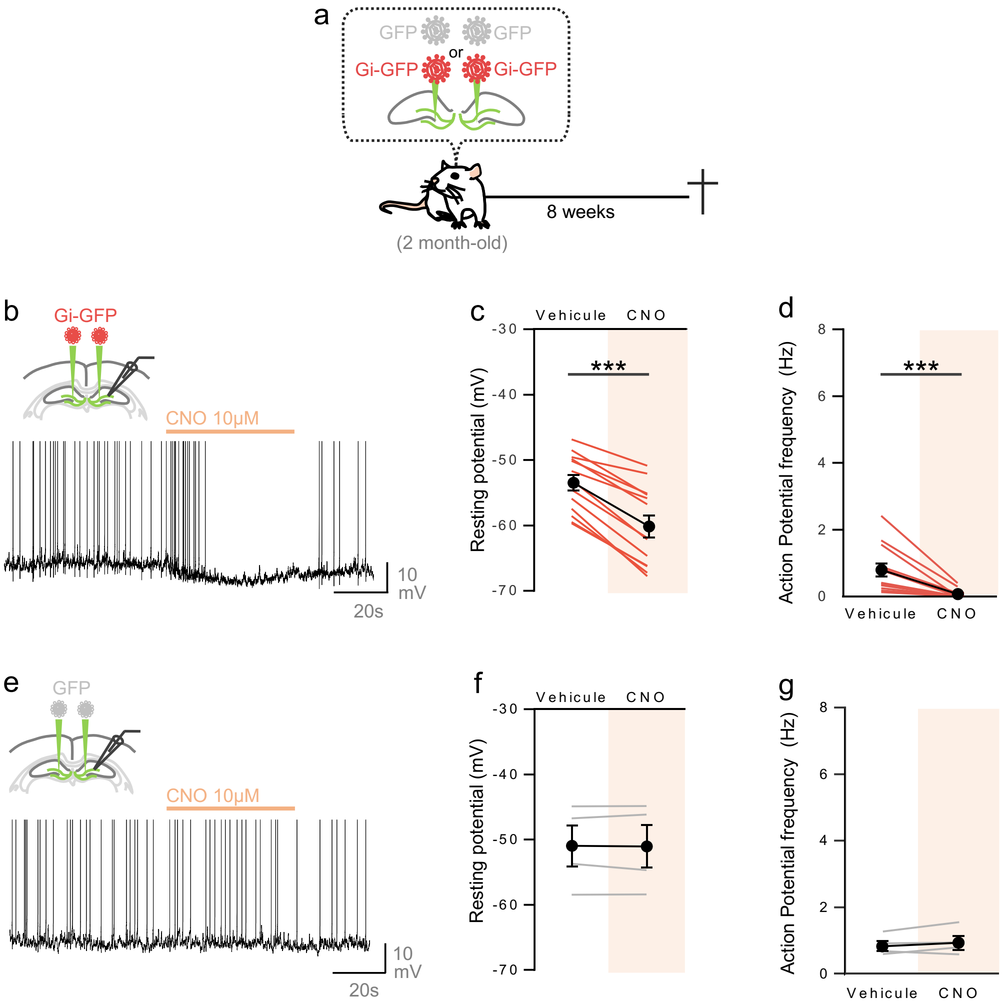

Fig S6. CNO application ex vivo quickly and reversibly inhibits Gi-GFP-RV transduced cells activity whereas it has no effect on the GFP-RV tranduced cells. a) Experimental protocol: Two-month old rats were injected bilaterally into the DG with the retrovirus Gi-GFP (n=4 rats, n=13 cells) or with its control, the GFP-RV (n=3 rats, n=4 cells). 8 weeks later they were sacrificed. **b)** Representative trace showing the inhibitory effect of CNO 10µM perfusion onto Gi-GFP RV infected cells. **c)** CNO hyperpolarizes Gi-GFP RV infected cells (Two-tailed T-test, t_{12}=10.66, ***p<0.001). **d)** CNO drastically reduces Gi-GFP RV tranduced cells action potential firing frequency (Two-tailed T-test, t_{12}=4.416 ***p=0.0008). **e)** Representative trace showing the absence of effect of CNO 10µM perfusion onto GFP RV infected cells. **f)** and **g)** CNO has no effect on GFP RV tranduced cells activity (Two-tailed T-test, t_3=0.2623 p=0.8101 and Two-tailed T-test, t_3=1.219 p=0.31). All data shown are mean ± s.e.m. For statistical details, see table S1.

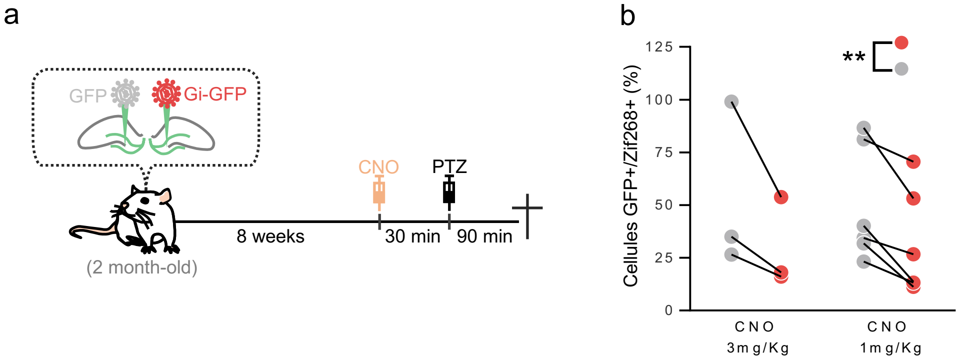

Fig S7. CNO injection in vivo inhibits Gi-GFP RV tranduced cells activity compared to GFP RV tranduced cells activity. a) Experimental protocol: Two-month old rats were injected into the right DG with the retrovirus Gi-GFP and with its control, the GFP RV into the left DG. 8 weeks later, one group received a 3mg/Kg CNO ip injection (n=3) and another group a 1mg/Kg CNO ip injection (n=6). 30 min later, both groups were injected wih a PTZ i.p injection. The rats were sacrificed 90 min later. **b)** Zif268 expression in GFP-IR cells. In both groups, percentage of expression was lower in the Gi-GFP cells compared to that of GFP cells (Tukey's test **p<0.01). For statistical details, see table S1.

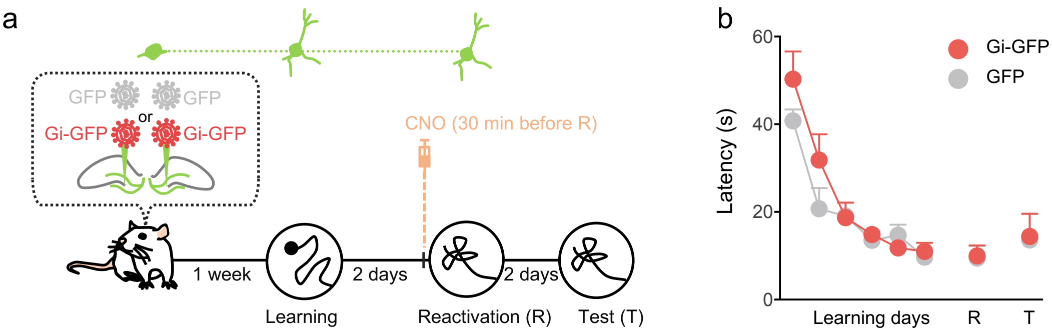

Fig S8. The effect of silencing immature adult-born neurons on recent memory reconsolidation. a) Experimental protocol: 2 month-old rats were injected with Gi-GFP RV (n=9) or its control GFP RV (n=8) one week before MWM training. Rats were trained for 6 days and memory was reactivated 2 days later. Thirty minutes before reactivation, CNO (1mg/Kg) was injected (i.p). Memory was tested 2 days later (Test). **b)** Latency to find the platform during training and to first cross the position of the platform during the test. There was no difference between groups. All data shown are mean ± s.e.m. For statistical details, see table S1.

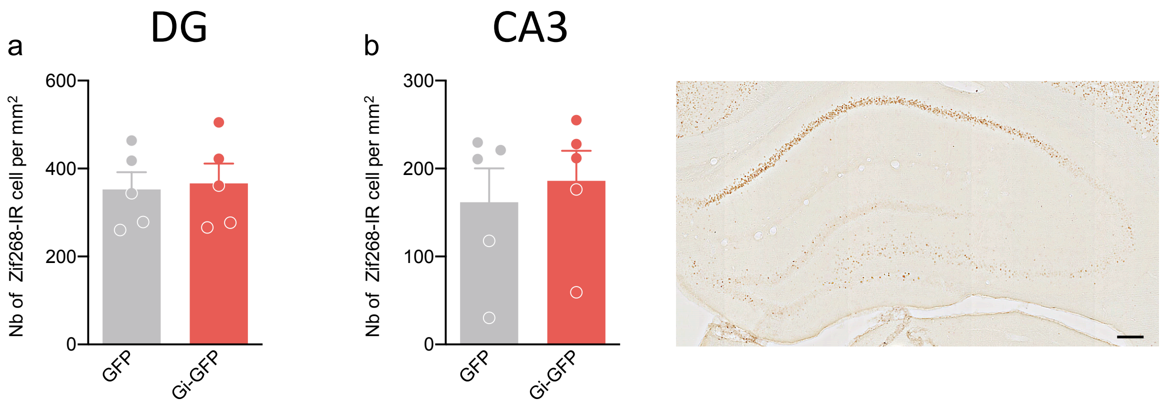

Fig S9. Silencing during reconsolidation, neurons that were immature at the time of learning, has no effect on global DG and CA3 activation. Zif268 expression in the whole Dentate Gyrus (DG) and CA3. The activation was similar among the trained groups (GFP n=5, Gi-GFP n=5 rats) both in **a)** DG (Two-tailed T-test, t_8=0.2269 p=0.8262) and **b)** CA3 (Two-tailed T-test, t_8=0.4719 p=0.6496). Illustration of Zif268-IR cells in the hippocampus. Bar scale 10µm. All data shown are mean ± s.e.m. For statistical details, see table S1.

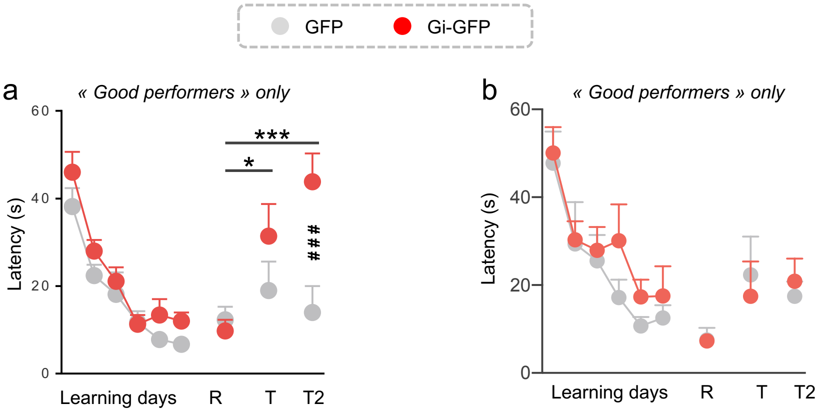

Fig S10. In good performers, silencing during reconsolidation, neurons that were immature at the time of learning, impairs long-term memory persistence whereas silencing neurons that were mature at the time of learning had no impact on memory. a) Latency to find the platform during training and to first cross the position of the platform during the reactivation and test trials only for the rats showing a good retention during the reactivation (less than 30sec to reach the platform position). Memory performances of Gi-GFP rats (n=8) were impaired compared to their performances at the reactivation trial as soon as the first test session (Tukey's test: ***p<0.05, ***p<0.001). Memory performances of Gi-GFP rats were impaired compared to those of GFP rats (n=8) at Test 2 (Tukey's test: ###p<0.001). **b)** Latency to find the platform during training and to first cross the position of the platform during the reactivation and test trials only for the rats showing a good retention during reactivation (less than 30sec to reach the platform position). Memory performances of Gi-GFP (n=9) rats and GFP (n=6) rats were similar. All data shown are mean ± s.e.m. For statistical details, see table S1.

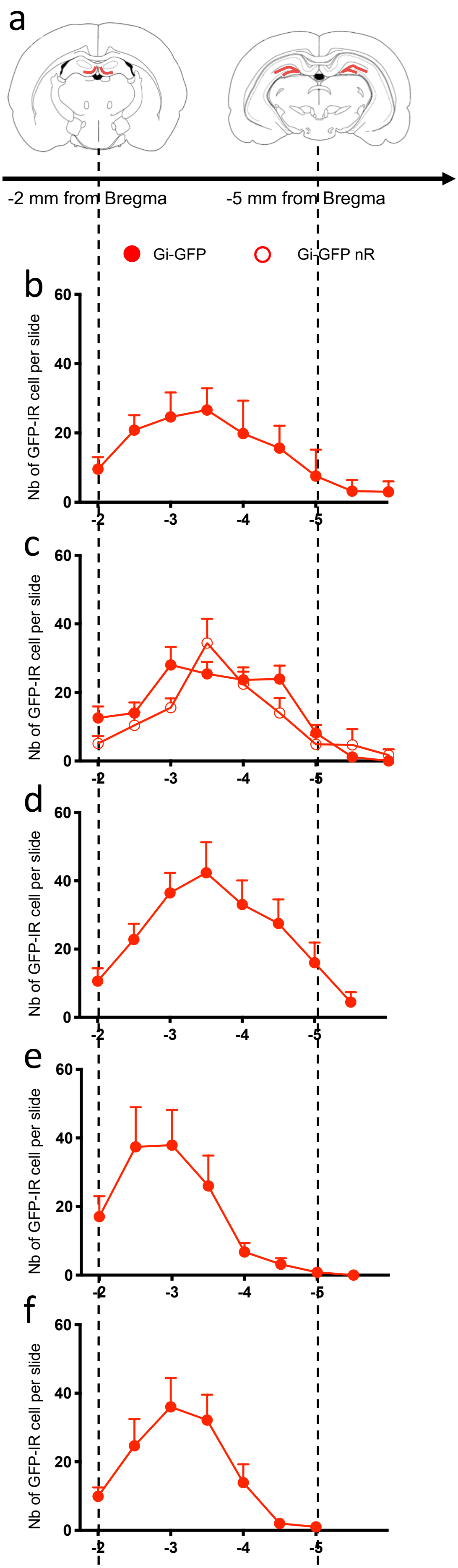

Fig S11. Septo temporal distribution of Gi-GFP labelled cells. **a)** Illustration of the rat brain according to the Paxinos Atlas. In red: dentate gyrus. Number of Gi-GFP-IR cells along the septo temporal axis in **b)** experiment on Fig 4 (n=5 rats), **c)** experiment on Fig5a (n=11 rats) , **d)** experiment on Fig 5d (n=11 rats), **e)** experiment on Fig 6 (n=12 rats). **f)** experiment on Fig 7 (n=12 rats). All data shown are mean ± s.e.m.

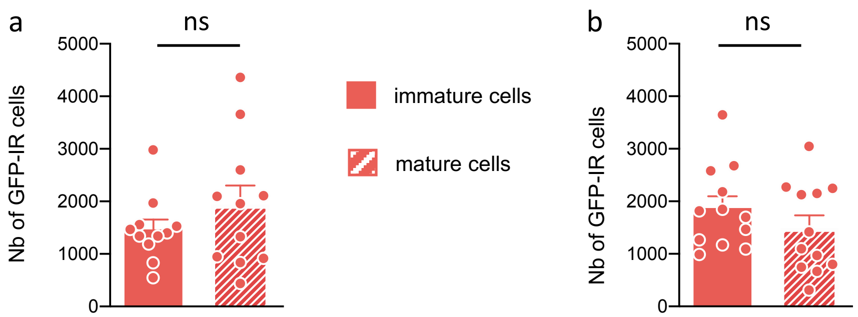

Fig S12. Number of transduced cells with the Gi-GFP retrovirus. a) The number of cells transduced with the Gi-GFP RV one week before learning in Fig 5a (called here immature cells, n=11 rats) was similar to the number of cells transduced with the Gi-GFP RV six weeks before learning in Fig 5b (here called mature cells, n=11 rats) (Two-tailed T-test, t_{20}=1.116 p=0.2778) . **b)** The number of cells transduced with the Gi-GFP RV one week before learning in Fig 6 (immature cell, n=12 rats) was similar to the number of cells transduced with the Gi-GFP RV six weeks before learning in Fig 7 (mature cells, n=12 rats) (Two-tailed T-test, t_{22}=1.139 p=0.2671). All data shown are mean ± s.e.m. For statistical details, see table S1.

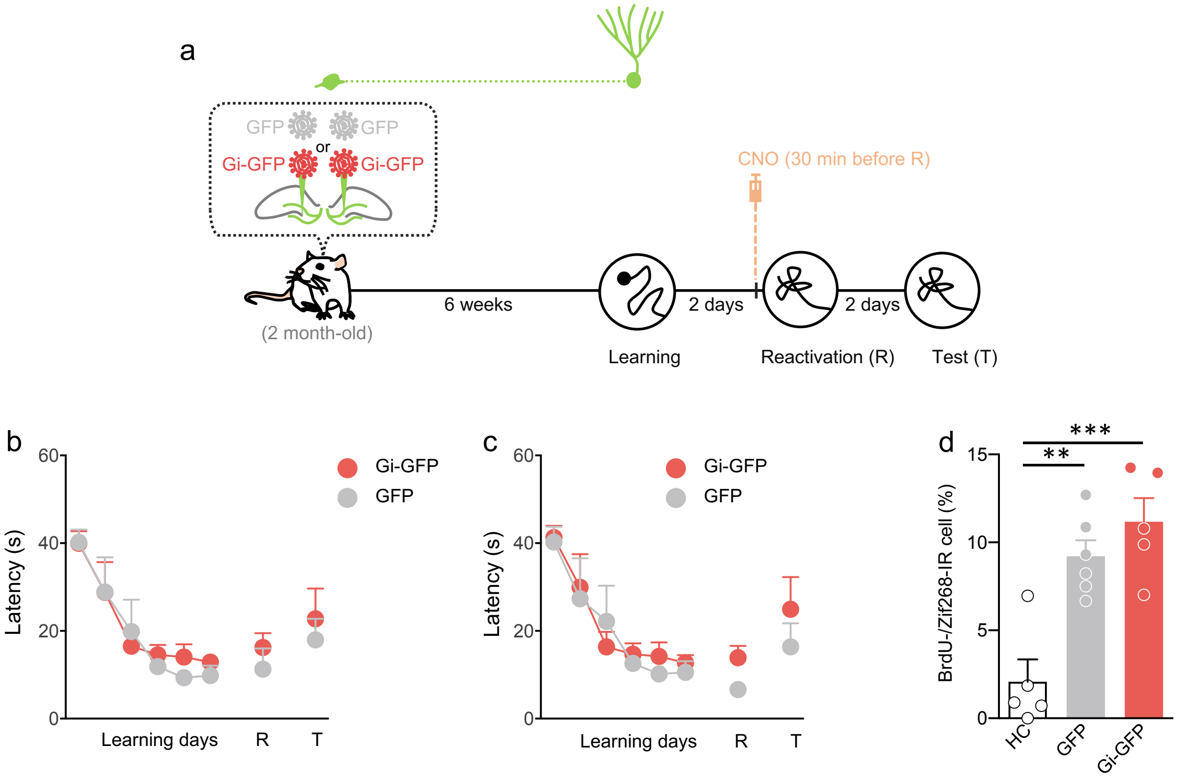

Fig S13. Silencing during recent reconsolidation, neurons that were 6 weeks old at the time of learning, has no impact on memory. a) Experimental protocol: 2 month-old rats were injected with Gi-GFP RV (n=10) or its control GFP RV (n=7) six week before MWM training. Rats were trained for 6 days and memory was reactivated 2 days later. 30 minutes before reactivation, rats were injected (i.p) with 1mg/Kg CNO. Memory was tested 2 days later (Test). **b)** Latency to find the platform during training and to first cross the position of the platform during the reactivation and test trial. Memory performances of Gi-GFP rats and GFP rats are similar. **c)** Latency to find the platform during training and to first cross the position of the platform during the reactivation and test trial only for the rats showing a good retention during reactivation (less than 30sec to reach the platform position). Memory performances of Gi-GFP (n=9) rats and GFP (n=6) rats are similar. **d)** Zif268 expression in BrdU-IR cells. Percentage of expression was higher in both the Gi and the GFP rats compared to that of control HC rats (n=5) (Tukey's test: ***$p<0.001$; **$p<0.01$). All data shown are mean ± s.e.m. For statistical details, see table S1.

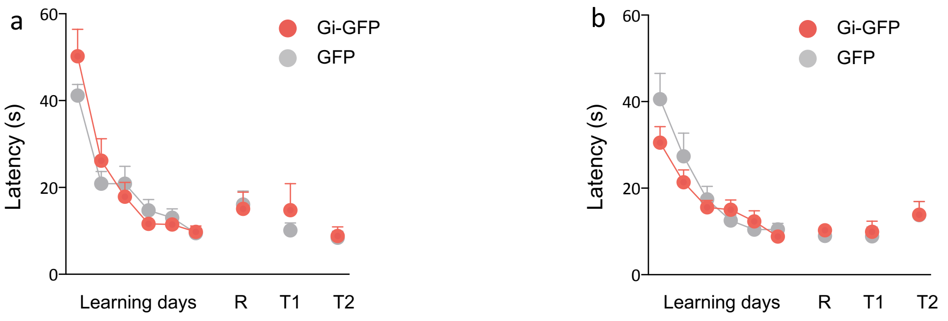

Fig S14. Effect of silencing adult-born neurons on latency to find the platform when memory is updated. a) There was no effect of silencing immature adult-born neurons on the latency to cross the platform at retrieval tests (Gi-GFP n=12 rats, GFP, n=12 rats). **b)** There was no effect of silencing mature adult-born neurons on the latency to cross the platform at retrieval tests (Gi-GFP n=12 rats, GFP, n=11 rats). All data shown are mean ± s.e.m. For statistical details, see table S1.

Figure	Panel	Statistical test	effects	statistic	p-value
Fig.1	b (D6-React-Test1) aCsf vs Ani	Two-way ANOVA (repeated measures)	No effect of group	F(1,15)=4.23	0.058
			Significant effect of time	F(2,30)=19.72	<0.0001
			Significant group x time interaction (Sidak and Turkey)	F(2,30)=6.92	0.003
	c	One-way ANOVA		F(2,18)=5.15	0.0169
	d	One-way ANOVA		F(3,22)=7.048	0,0017
Fig.2	b	One-way ANOVA		F(3,39)=10.98	<0.0001
	d			F(3,39)=0.9469	0.4274
Fig 3	b (d6-T)	Two-way ANOVA (repeated measures)	Significant effect of group	F(3,30)=3.201	0.0373
			Significant effect of time	F(1,30)=5.080	0.0317
			Significant group x time interaction (Sidak and Turkey)	F(3,30)=3.022	0.0450
	d (d6-T)	Two-way ANOVA (repeated measures)	No effect of group	F(1,15)=0.2	0.6551
			No effect of time	F(1,15)=0.5	0.4549
			No group x time interraction	F(1,15)=0.4	0.5313
	e	Unpaired T-test		T(9)=1.604	0.1433
	f	Unpaired T-test		T(9)=3.831	0.0040
Fig 4	b (D6-React-Test) GFP vs Gi-GFP	Two-way ANOVA (repeated measures)	Significant effect of group	F(1,12)=7.75	0.0165
			no effect of time	F(2,24)=2.822	0.0793
			Significant group x time interaction (Sidak and Turkey)	F(2,24)=3.796	0.0369
	c	One-way ANOVA		F(2,12)=39.46	<0.0001
	d	One-way ANOVA		F(2,12)=6.718	0.0110
	f	One-way ANOVA		F(2,12)=35.19	<0.0001
Fig.5	b (D6-React-Test1&2) GFP vs Gi-GFP	Two-way ANOVA (repeated measures)	No effect of group	F (1, 19) = 2.520	0.1289
			Significant effect of time	F (3, 57) = 7.348	0.0003
			Significant group x time interaction	F (3, 57) = 2.835	0.0461
	c	One-way ANOVA		F (2, 28) = 4.840	0.0157
	e (D6-React-Test1&2)	Two-way ANOVA (repeated measures)	No effect of group	F (1, 15) = 0.1687	0.6870
			No effect of time	F (3, 45) = 0.8603	0.4686
			No group x time interaction	F(3,45)=0.5796	0.6314

Fig.6	b	Three-way ANOVA (repeated measures)	Significant effect of time	F(2,44)=7.0272	0.0022
			Significant group x time interaction	F(2,44)=3.7816	0.0305
			Significant effect of zone	F(1,22)=29.2115	<0.0001
			No group x zone interaction	F(1,22)=2.6418	0.1183
			Significant time x zone interaction	F(2,44)=7.1315	0.0021
			Significant time x zone x group interaction (Newman-Keuls)	F(2,44)=3.7925	0.0302
	c	Three-way ANOVA (repeated measures)	No effect of group	F(1,22)=0.4351	0.5163
			Significant effect of time	F(2,44)=4.7199	0.0139
			Significant group x time interaction	F(2,44)=2.5563	0.0890
			Significant effect of zone	F(1,22)=26.7146	<0.0001
			No group x zone interaction	F(1,22)=1.5164	0.2312
			Significant time x zone interaction	F(2,44)=5.7216	0.2312
			Significant time x zone x group interaction (Newman-Keuls)	F(2,44)=3.2233	0.0494
	f	Two-way ANOVA (repeated measures)	No effect of group	F(1,22)=0.3720	0.5481
			Significant effect of time	F(2,44)=5.375	0.0082
			Significant group x time interaction (Sidak and Turkey)	F(2,44)=3.517	0.0383
	g		No effect of group	F(1,22)=0.5051	0.4847
			No effect of time	F(2,44)=2.279	0.1143
			Significant group x time interaction (Sidak and Turkey)	F(2,44)=4.170	0.0220
Fig.7	b	Three-way ANOVA (repeated measures)	No effect of group	F(1,18)=0.2600	0.6163
			Significant effect of time	F(2,36)=7.4450	0.0019
			No group x time interaction	F(2,36)=0.3891	0.6805
			Significant effect of zone	F(1,18)=36.8225	<0.0001
			No group x zone interaction	F(1,18)=0.4405	0.5153
			Significant time x zone interaction (Newman-Keuls)	F(2,36)=8.9414	0.0007
			No time x zone x group interaction	F(2,36)=0.1693	0.8449

	c	Three-way ANOVA (repeated measures)	No effect of group	F(1,18)=0.4945	0.4909
			Significant effect of time	F(2,36)=5.2411	0.0100
			No group x time interaction	F(2,36)=0.3267	0.7234
			Significant effect of zone	F(1,18)=37.9301	<0.0001
			No group x zone interaction	F(1,18)=1.4589	0.2427
			Significant time x zone interaction (Newman-Keuls)	F(2,36)=9.9318	0.0004
			No time x zone x group interaction	F(2,36)=0.2028	0.8117
	e	Two-way ANOVA (repeated measures)	No effect of group	F(1,18)=0.06305	0.8046
			Significant effect of time (Turkey)	F(2,36)=10.90	0.0002
			No group x time interaction	F(2,36)=0.7099	0.4984
	f	Two-way ANOVA (repeated measures)	No effect of group	F(1,18)=0.06249	0.8054
			Significant effect of time (Turkey)	F(2,36)=6.734	0.0033
			No group x time interaction	F(2,36)=1.501	0.2365
**Fig.S1**	**a**	One-way ANOVA		F(2,18)=0.6892	0.5117
	**b**	One-way ANOVA		F(2,18)=0.02511	0.9752
**Fig.S2**	**a** (D6-React-Test) aCsf vs Ani	Two-way ANOVA (repeated measures)	No effect of group	F(1,22)=2.010	0.1703
			No effect of time	F(2,44)=1.4823	0.6206
			Significant group x time interaction (Sidak and Turkey)	F(2,44)=4.459	0.0173
	**b**	One-way ANOVA		F (2, 27) = 1.369	0.2714
**Fig.S3**	**b**	One-way ANOVA		F(2,10)=1.699	0.2316
**Fig.S4**	**b** (D6-React-Test) aCsf vs Ani	Two-way ANOVA (repeated measures)	No effect of group	F (1, 11) = 2.546	0.1389
			Significant effect of time	F (2, 22) = 7.908	0.0026
			Significant group x time interaction (Sidak and Turkey)	F (2, 22) = 7.373	0.0035
	**c**	One-way ANOVA		F (3, 24) = 5.700	0.0043
	**d**	One-way ANOVA		F (3, 24) = 1.99	0.14
	**e**	One-way ANOVA		F (3, 17) = 0.0374	0.9634
**Fig.S5**	**b**	T-test		T(6)=1.028	0.3437
**Fig.S6**	**c**	Paired T-test		T(12)=10.66	<0.0001
	**d**			T(12)=4.416	0.0008
	**f**			T(3)=0.2623	0.8101

	**g**			T(3)=1.219	0.3100
**Fig S7**	**b**	Two-way ANOVA (repeated measures)	No effect of CNO dose	F (1, 7) = 0.0024	0.9617
			Significant effect of RV	F (1, 7) = 20.45	0.0027
			No CNO dose x RV interaction	F (1, 7) = 0.4116	0.5416
**Fig S8**	**b** (D6-React-Test) GFP vs Gi-GFP	Two-way ANOVA (repeated measures)	No effect of group	F(1,15)=0.04246	0.8395
			No effect of time	F(2,30)=1.424	0.2566
			No group x time interaction	F(2,30)=0.0097	0.9903
**Fig S9**	**a**	Unpaired T-test		T(8)=0.2269	0.8262
	**b**	Unpaired T-test		T(8)=0.4719	0.6496
**Fig S10**	**a** (D6-React-Test1&2)	Two-way ANOVA (repeated measures)	Significant effect of group	F (1, 14) = 14.16	0.0021
			Significant effect of time	F (3, 42) = 7.350	0.0005
			Significant group x time interaction (Sidak and Turkey)	F (3, 42) = 3.621	0.0206
	**b** (D6-React-Test1&2)	Two-way ANOVA (repeated measures)	No effect of group	F(1,13)=0.02206	0.8842
			No effect of time	F(3,39)=2.577	0.0675
			No group x time interaction	F(3,39)=0.3770	0.7701
**Fig S12**	**a**	Unpaired T-test		T(20)=1.116	0.2778
	**b**	Unpaired T-test		T(22)=1.139	0.2671
**Fig S13**	**b** (D6-React-Test) GFP vs Gi-GFP	Two-way ANOVA (repeated measures)	no effect of group	F(1,15)=1.088	0.3134
			no effect of time	F(2,30)=2.341	0.1136
			no group x time interaction	F(2,30)=0.02932	0.9711
	**c** (D6-React-Test) GFP vs Gi-GFP	Two-way ANOVA (repeated measures)	no effect of group	F(1,13)=1.896	0.1918
			significant effect of time	F(2,26)=3.908	0.0328
			no group x time interaction	F(2,26)=0.3476	0.7096
	**d**	One-way ANOVA		F(2,13)=16,16	0.0003
**Fig S14**	**a** (D6-React-Test 1&2) GFP vs Gi-GFP	Two-way ANOVA (repeated measures)	no effect of group	F(1,22)=0.1342	0.7176
			Significant effect of time	F(3,66)=2.922	0.0404
			no group x time interaction	F(3,36)=0.4517	0.7170
			no effect of group	F(1,21)=0.01411	0.9066

	**b** (D6-React-Test 1&2) GFP vs Gi-GFP	Two-way ANOVA (repeated measures)	significant effect of time	F(3,63)=2.404	0.0758
			no group x time interaction	F(3,63)=0.2366	0.8705

Table S1: Statistical analyses for each figure: From left to right : figure number, panel, statistical test used, effects analysed, F or T value for Anova or T test, value of probability (p) for significance.

PCR primers	Forward (5'-3')	TATATGGATCCATGTACCCATACGATGTTCCAGATTACGCTGCCAAC
	Reverse (5'-3')	TATATGGATCCCTACCTGGCAGTGCCGATGT
Sequencing primers	Forward (5'-3')	CCTTCTCCCTCTCCAGCCT
	Reverse (5'-3')	CTCAAGAGCCCACTAATGAAG

Table S2: PCR primers used to clone HA-hM4D(G_i) into CAG-IRES-GFP and primers used to sequence the resulting construct CAG-G_i-IRES-GFP.
