# Supplementary material for: Adult-born neurons immature during learning are necessary for remote memory reconsolidation in rats
Source: Nat Commun. 2021 Mar 19;12:1778. doi: 10.1038/s41467-021-22069-4 (PMC7979763; doi:10.1038/s41467-021-22069-4)
Supplement: Supplementary file 3 — Reporting Summary [file 41467_2021_22069_MOESM3_ESM.pdf]

nature research

Corresponding author(s): sophie TRONEL

Last updated by author(s): 17/02/2021

Reporting Summary

Nature Research wishes to improve the reproducibility of the work that we publish. This form provides structure for consistency and transparency in reporting. For further information on Nature Research policies, see Authors & Referees and the Editorial Policy Checklist .

Statistics

For all statistical analyses, confirm that the following items are present in the figure legend, table legend, main text, or Methods section.

n/a	Confirmed	
☐	☒	The exact sample size (*n*) for each experimental group/condition, given as a discrete number and unit of measurement
☒	☐	A statement on whether measurements were taken from distinct samples or whether the same sample was measured repeatedly
☐	☒	The statistical test(s) used AND whether they are one- or two-sided<br>*Only common tests should be described solely by name; describe more complex techniques in the Methods section.*
☒	☐	A description of all covariates tested
☐	☒	A description of any assumptions or corrections, such as tests of normality and adjustment for multiple comparisons
☐	☒	A full description of the statistical parameters including central tendency (e.g. means) or other basic estimates (e.g. regression coefficient) AND variation (e.g. standard deviation) or associated estimates of uncertainty (e.g. confidence intervals)
☐	☒	For null hypothesis testing, the test statistic (e.g. *F*, *t*, *r*) with confidence intervals, effect sizes, degrees of freedom and *P* value noted<br>*Give P values as exact values whenever suitable.*
☒	☐	For Bayesian analysis, information on the choice of priors and Markov chain Monte Carlo settings
☒	☐	For hierarchical and complex designs, identification of the appropriate level for tests and full reporting of outcomes
☒	☐	Estimates of effect sizes (e.g. Cohen's *d*, Pearson's *r*), indicating how they were calculated

Our web collection on statistics for biologists contains articles on many of the points above.

Software and code

Policy information about availability of computer code

Data collection	Viewpoint tracking system (V 3,9,0,22) was used for water maze behavioral experiments, POLY file from Imetronic (V1) was used to study navigational strategy. Mercator software (Explora Nova, V4.0.0.0) was used for the measurement of Zif268 positive neurons. Clampfit software pClamp 10, Molecular devices, UK, V.10.7 was used for electrophysiological analyes
Data analysis	GraphPad Prism 6 and 8 were used to analyse data, generate graph and statistics

For manuscripts utilizing custom algorithms or software that are central to the research but not yet described in published literature, software must be made available to editors/reviewers. We strongly encourage code deposition in a community repository (e.g. GitHub). See the Nature Research guidelines for submitting code & software for further information.

Data

Policy information about availability of data

All manuscripts must include a data availability statement. This statement should provide the following information, where applicable:
- Accession codes, unique identifiers, or web links for publicly available datasets
- A list of figures that have associated raw data
- A description of any restrictions on data availability

The data that support the findings of this study are available in the source file

Field-specific reporting

Please select the one below that is the best fit for your research. If you are not sure, read the appropriate sections before making your selection.

Life sciences study design

All studies must disclose on these points even when the disclosure is negative.

Sample size	No statistical methods were used to predetermine sample sizes. Over the 15 years of experience in using the water maze in rats we found that 8 rats per group is enough to reach statistical differences. To face the variability of the surgery, we increased the sample size to 10-12 rats per group. (Tronel et al, 2010, PNAS; Tronel et al, 2012, Hippocampus)
Data exclusions	Animals in which histological examination showed that cannulae or viral targeting were in the incorrect location were excluded from analysis after behavioural tests. The exclusion criteria was pre-established.
Replication	Experiment on the effect of anisomycin on memory reconsolidation was replicated 3 times (shown in Fig 1 and S2). Experiment on the effect of silencing immature neurons (Fig3) was replicated three time. All other experiment were replicated once. All attempts at replication were successful.
Randomization	All rats were assigned to different experimental conditions randomly
Blinding	investigators were blind to group allocation in both behavioral experiments and cell counting.

Reporting for specific materials, systems and methods

We require information from authors about some types of materials, experimental systems and methods used in many studies. Here, indicate whether each material, system or method listed is relevant to your study. If you are not sure if a list item applies to your research, read the appropriate section before selecting a response.

Materials & experimental systems

n/a	Involved in the study	
☐	☒	Antibodies
☐	☒	Eukaryotic cell lines
☒	☐	Palaeontology
☐	☒	Animals and other organisms
☒	☐	Human research participants
☒	☐	Clinical data

Methods

n/a	Involved in the study	
☒	☐	ChIP-seq
☒	☐	Flow cytometry
☒	☐	MRI-based neuroimaging

Antibodies

Antibodies used

GFP chicken primary antibody Abcam Cambridge #Ab 13970
BrdU Mouse primary antibody Dako Agilent, Santa Clara US #M0744
IdU Mouse primary antibody BD #347580
CldU rat primary antibody Accurate C&S CO #OBT0030
Zif 268 Rabbit primary antibody Santa Cruz Biotechnology #SC-189
Biotinylated goat anti chicken , Jackson Immunoresearch, #103-065-155
Horse anti mouse, Vector labs, Peterborough #BA-2001
Goat anti rat, Vector labs, Peterborough #BA-9400
Cy3goat anti rat , Jackson Immunoresearch, #112-165-062
Cy3goat anti mouse , Jackson Immunoresearch, 115-165-003
Alexa 488 goat anti rabbit , invitrogen #A11008
Cy5goat anti rabbit, Chemicon ## A10523
Dcx Rabbit primary antibody , Sigma # D 9818
BrdU Mouse primary Antibody, Accurate C&S CO #OBT0030
Calbindin goat primary antibody, Santa Cruz Biotechnology #SC7691

Validation

GFP chicken primary antibody Abcam Cambridge #Ab 13970 validated for IHC : https://www.abcam.com/gfp-antibody-ab13970.html
BrdU Mouse primary antibody Dako Agilent, Santa Clara US #M0744: https://www.agilent.com/cs/library/packageinsert/public/109731002.PDF
IdU Mouse primary antibody BD #347580: https://www.citeab.com/antibodies/2414712-347580-purified-mouse-anti-brdu
CldU and BrdU rat primary antibody Accurate C&S CO #OBT0030: https://www.bio-rad-antibodies.com/monoclonal/brdu-antibody-bu20a-mca2483.html?f=purified
Zif 268 Rabbit primary antibody Santa Cruz Biotechnology #SC-189: https://www.citeab.com/antibodies/790874-sc-189-egr-1-

antibody-c-19
Biotinylated goat anti chicken , Jackson Immunoresearch, #103-065-155: https://www.citeab.com/antibodies/2035704-103-065-155-biotin-sp-affinipure-goat-anti-chicken-i
Horse anti mouse, Vector labs, Peterborough #BA-2001: https://www.citeab.com/antibodies/3379372-ba-2001-biotinylated-horse-anti-mouse-igg-antibody
Goat anti rat, Vector labs, Peterborough #BA-9400, https://vectorlabs.com/biotinylated-goat-anti-rat-igg-antibody.html
Cy3goat anti rat , Jackson Immunoresearch, #112-165-062, https://www.jacksonimmuno.com/catalog/products/112-165-062
Cy3goat anti mouse , Jackson Immunoresearch, 115-165-003, https://www.jacksonimmuno.com/catalog/products/115-165-003
Alexa 488 goat anti rabbit , invitrogen #A11008, https://www.thermofisher.com/antibody/product/Goat-anti-Rabbit-IgG-H-L-Cross-Adsorbed-Secondary-Antibody-Polyclonal/A-11008
A647 Donkey anti Goat, Jackson Immunoresearch, #705-605-147, https://www.jacksonimmuno.com/catalog/products/705-605-147
Cy5goat anti rabbit, Chemicon ## A10523, https://www.fishersci.com/shop/products/anti-rabbit-igg-h-l-cy-5-conjugated-polyclonal-thermo-scientific-novex/A10523
Dcx Rabbit primary antibody , Sigma # D 9818, https://www.sigmaaldrich.com/catalog/product/sigma/d9818?lang=fr&region=FR
Calbindin goat primary antibody , Santa Cruz Biotechnology #SC7691, https://www.scbt.com/fr/p/calbindin-d28k-antibody-c-20?requestFrom=search

Eukaryotic cell lines

Policy information about cell lines

Cell line source(s)	The 293GPG cell line was provided by Dr Dieter Chichung Lie
Authentication	The 293GPG cell line was generated by Ory DS et al . PNAS 1996
Mycoplasma contamination	cell lines were tested negative for mycoplasma contamination
Commonly misidentified lines (See ICLAC register)	N/A

Animals and other organisms

Policy information about studies involving animals; ARRIVE guidelines recommended for reporting animal research

Laboratory animals	All rats were Sprague Dawleys OFA from Charles River. Male were delivered at 2 month-old at the beginning of the experiments. Ten Pregnant females were delived at the age of 3 month-old.
Wild animals	This study did not involve wild animals.
Field-collected samples	This study did not involve samples collected from the field.
Ethics oversight	all experiments were performed in accordance with the recommendations of the European Union (2010/63/UE) and were approved by the ethical comittee of the Unviversity of Bordeaux (#Dir 1367 and #DIR23375)

Note that full information on the approval of the study protocol must also be provided in the manuscript.
